# Female begging calls reflect nutritional need of nestlings in the hen harrier *Circus cyaneus*

**DOI:** 10.1186/s12862-017-0986-z

**Published:** 2017-06-19

**Authors:** Steve Redpath, Alex Thompson, Arjun Amar

**Affiliations:** 1 Institute of Biological and Environmental Science, Aberdeen University, Zoology Building, Tillydrone Av, Aberdeen, AB24 2TZ UK; 2 FitzPatrick Institute of African Ornithology, DST/NRF Centre of Excellence, University of Cape Town, Rondebosch, 7701 South Africa; 3 Department of Ecology, Swedish University of Agricultural Science, Grimso Wildlife Research Station, -730 91 Riddarhyttan, SE Sweden

**Keywords:** Begging behaviour, Nestlings, Provisioning behaviour, Breeder need, Offspring need, Raptors, Hen harrier, Sexual conflict

## Abstract

**Background:**

Most birds exhibit bi-parental care with both sexes providing food for their young. Nestling signal food needs through begging. However, for some species, males rarely visit the nest, so have limited opportunity for gaining information directly from the chicks. Instead, females beg when males deliver food. We tested whether this calling signalled nutritional need and specifically the needs of the female (*Breeder Need hypothesis*) or that of their chicks (*Offspring Need hypothesis*).

**Results:**

We observed begging and provisioning rates at 42 nests of hen harrier (*Circus cyaneus*) in Scotland, explored the factors associated with variation in begging rate and the relationship between begging and provisioning. We also tested the impact of food on begging and provisioning through a feeding experiment. Female begging rate increased up to a chick age of 3 weeks and then tailed off. In addition, begging increased when broods were large.

**Conclusions:**

Our data provided support for the *Offspring Need hypothesis*. At nests where *adlib* food was provided females reduced their begging rate. These patterns suggested that female begging was an honest signal of need. However, begging continued even with *adlib* food and was only weakly associated with greater provisioning by males, suggesting that these calls may also play an additional role, possibly reflecting sexual or parent-offspring conflict.

## Background

Breeding birds have to decide how much time to devote to foraging versus a range of other behaviours, such as protecting their young against adverse weather or predators. These decisions are complicated when the two parents are foraging independently as each adult needs a way of assessing how hungry their chicks are. Fortunately, chicks are able to signal their hunger to their parents by making loud begging calls, and studies have suggested that these calls are reliable indicators of chick nutritional needs [10], with adults varying their provisioning rate in response to variation in chick begging rates [19, 11, 15, 41]. This honesty may, however, be context dependent, and begging might be less honest when the potential for conflict between chicks and parents is high [34].

In systems with bi-parental care, each adult can gain an assessment of chick needs during their visits to the nest. However, in some birds such as raptors and parrots it is common for one sex to rarely, if ever, visit the nest during the chick-rearing period. Instead, the female attends to the chicks and feeds them, whilst the male either drops prey on a perch nearby (eg goshawk *Accipiter gentilis*, peregrine *Falco peregrinus*, or sparrowhawk *Accipiter nisus*), or passes the food to the female in the air (eg harriers *Circus* spp.). The males of these species therefore gain little direct information about chick condition on which to base their foraging decisions. In such cases, males are dependent on females to provide information on chick need.

Begging calls by adult females before and during the breeding period occurs in a variety of species [6, 9, 23]. During the pre-laying and incubation stages evidence suggests that female calls signal both fertility to potential extra-pair partners and their nutritional need [6, 9, 23]. During the nestling stage, as Ellis et al. [9] pointed out, calls might reflect chick need (*Offspring Need Hypothesis*) or female need (*Breeder Need Hypothesis*). In either case, given the potential risks of calls attracting predators, we would expect female begging calls to honestly reflect the needs of the offspring or the female, and for begging call rate to be positively associated with male provisioning [23]. However, as parental investment in offspring may be costly [7, 20], females may also use their begging calls to encourage males to provide more food, so that females then invest their time in chick protection. In other words, a potential conflict may exist between the parents over how they should allocate their time to their current brood [3, 12–14].

In the hen harrier *Circus cyaneus* the “twiss-you” call (hereafter called the begging call) is uttered repeatedly by the female and is directed at the male, usually during and after food provisioning [44]. This call is loud and can be heard by humans up to 1 km away. Male hen harriers rarely visit the nest, especially when the chicks are small, but instead call to the female and pass the food to her away from the young. The time females spend at the nest declines rapidly over the first 3 weeks and then remains low until fledging [17]. This is reflected in their provisioning rates, which increase over the first three weeks then stay constant [31]. Studies of weight loss in breeding female raptors suggest that weight is maintained during incubation, but is subsequently lost during the nestling period [25, 43]. This pattern reflects the fact that females preferentially feed their offspring, rather than themselves [25]. Given this pattern, if the *Breeder Need Hypothesis* was true, we would expect female begging rate to increase over the course of the nestling period as their weight loss increased. In contrast, if the *Offspring Need Hypothesis* was true, we would expect the rate of begging to reflect growth rates and food intake needs of the chicks and therefore to be lowest at hatching and greatest for middle nestling ages [26, 28, 29].

To test these hypotheses and explore patterns in female begging, we first confirmed the relationship between the food intake rate of chicks and nestling age; second, we examined how female begging varied in relation to the age of chicks and brood size; third, we examined the relationship between female begging rate and male provisioning to explore whether males can adjust their provisioning in relation to female begging. Lastly, we manipulated food at some nests over two years to test whether, as expected, increased food led to decreases in begging and male provisioning rate.

## Methods

Female begging was recorded at 42 breeding hen harrier nests from 1994 to 1999 on Langholm estate in south-west Scotland (55^o^ 10′N, 2^o^ 58′W). Harrier nests were watched during the breeding season from hides set 6 to 10 m away from the nest. No nests were watched in 1997. The hides were set up once the first egg had hatched and nests were generally monitored up to the point of chick fledging (Table 1). We recorded begging behaviour at all nests during 667 watches, typically lasting 5–6 h (mean ± sd = 5.7 ± 0.6 h). During each watch we recorded the age (in weeks) and number of harrier chicks, the number of food deliveries and the number of begging calls given by females. Males provided most of the food during the first two weeks [31], with the females tending to stay close to the nest. In 1994/95 we recorded feeding patterns at nests (unpublished data). Of the 24,640 morsels of food seen consumed, the chicks ate 93.6%, implying that the female preferentially fed her offspring as recorded for sparrowhawks [25].

**Table 1:** Summary of data from 42 hen harrier nests, watched during years 1994–1999

Year	Nest number	Chick ages (weeks)	Number of chicks	Fed / Unfed	Time watched (hrs)	Number of Bouts	Number of calls
1994	1	2–6	5–4	U	84.03	73	882
1994	2	1–6	4–3	U	105.97	45	817
1994	3	1–3	5–2	U	31.61	13	98
1994	4	1–6	4–2	U	73.46	52	1592
1994	5	2–6	6–4	U	107.32	63	2479
1994	6	2–6	3	U	76.38	23	501
1994	7	1–4	3	U	66.87	54	1682
1995	8	1–6	5–4	U	99.64	107	1812
1995	9	1–6	4–3	U	105.51	33	708
1995	10	1–6	4	U	91.8	117	2721
1995	11	2–5	4	U	115.82	86	2692
1995	12	1–5	3	U	86.14	114	2260
1995	13	1–5	5–3	U	114.94	114	2647
1996	14	1–6	4–2	U	152.22	99	2244
1996	15	1–5	4–3	U	142.22	112	1396
1996	16	1–5	6–4	U	114.14	88	3645
1996	17	1–5	4–3	U	101.16	117	3089
1996	18	1–6	3–2	U	132.81	108	4039
1998	19	1–6	5–4	U	87.09	79	2837
1998	20	2–5	3	U	82.95	98	3602
1998	21	1–5	5–2	U	77.67	56	1470
1998	22	1–5	5–4	U	78.34	85	2603
1998	23	3–6	4–2	U	68.58	45	1005
1998	24	2–6	5–3	U	77.5	78	1918
1998	25	1–5	4–3	F	70.35	83	1206
1998	26	2–5	4–3	F	57.43	41	396
1998	27	2–5	3–2	F	58.5	35	1503
1998	28	1–5	5	F	53.91	61	846
1998	29	2–5	2	F	59.5	66	1047
1998	30	2–5	3	F	70.48	29	520
1998	31	1–5	5	F	95.33	115	1780
1998	32	1–5	4	F	63.99	56	704
1998	33	2–5	6–4	F	86.77	61	1270
1999	34	1–5	2	U	78.41	66	829
1999	35	1–5	4–2	U	113.66	66	1034
1999	36	1–5	2	U	77.84	67	1871
1999	37	2–5	3	U	101.58	78	3027
1999	38	1–5	5–4	F	95.5	27	187
1999	39	1–5	2	F	112.84	56	735
1999	40	1–5	4–3	F	125.25	78	602
1999	41	1–5	4–2	F	112.09	61	1174
1999	42	2–5	3	F	101.58	46	496

The table shows the range of chick ages at each nest during watches, the number of chicks, showing decline due to chick mortality, the total number of hours each nest was watched for, the number of bouts of begging and the total number of begging calls recorded

In 1998 and 1999, 14 of the 42 nests were provided with *adlib* food (dead day old cockerel chick *Gallus gallus domesticus* and white lab rats *Rattus norvegicus* at a ratio of approximately 3:1) from hatching to fledging in a field experiment to test the effect of feeding on predation rates on red grouse chicks [1, 32]. In the same years, 10 nests were also monitored as controls. Perches were erected on average 9 ± 1 m from the nests in the fed treatment. These were visited daily and fresh food was put out. The estimated food consumed by the fed chicks ranged from 40 g per day at one week old to 185 g per day at 5 weeks old [39]. The amount of food put out per day therefore reflected these amounts. In total, 10,568 food items were placed on the perches, of which birds removed 8332 (79%) items [32]. Of the rats and chicks seen delivered to nests during watches, females delivered 40 per 100 h, compared to 4 by their males. All uneaten food was removed and disposed of the following day. The availability of food greatly reduced the delivery rate of wild prey by females, but not males [32].

Of the breeding adults, 9 males and 8 females were tagged. Two of these tagged males and three of the females were observed during nest watches in different years, so there was some pseudo-replication in the data. However, as the majority of birds were not tagged, it was not possible to know the extent of this potential problem.

Female harriers usually left the nest when the male approached with food and broadcast the “twiss-you” call to the male until returning to the nest with the prey [8]. After feeding, females sometimes left the nest to drop prey remains, collect nest material, rest away from the chicks, fly around the nest territory or hunt. In such cases if the male was still present in the area she would call until he disappeared. Usually calls were associated with a prey delivery, but sometimes the female would see the male and call at him with no exchange of food. Very occasionally, if the female was not present when the male arrived, he would drop prey at the nest and quickly leave without any female vocalisation. For the purposes of our analyses, rather than recording calls per prey delivery we recorded calls per “bout” of female calls, where a new bout was recorded if it occurred at least 10 min after the last female call. During each watch we recorded both the number of bouts and the number of individual calls per bout. These two measures were then used to derive an average calls per bout per watch and this was the unit for analysis.

Prey delivery rates (g/h) were calculated using the same approach as Leckie et al. [17] and Redpath et al. [33]. Briefly, we assigned a weight to each prey item brought to nests, based on estimates from the literature [29, 36] or our own measurements. Prey items that could not be identified were invariably small items, which were rapidly eaten and were estimated at 20 g. As harriers are sometimes polygynous with males breeding with two or more females, and secondary females may receive less prey from their males [33], we also recorded the status of each female as monogamous, an alpha female of a bigamous male or a beta female of a bigamous male.

Fieldwork was carried out under licence from Scottish Natural Heritage.

### Statistical analysis

All analyses were conducted in R version 3.2.3 [30]. To explore how nutritional need of chicks at nests varied with age, we estimated the amount of food (g) delivered to the 14 nests where food was provided *adlib*. We assumed that food delivered reflected nestling requirements, free from any constraints imposed by other factors such as prey capture rates. We used a Linear Mixed Model with a Gaussian distribution, with biomass of food (g) delivered to the nest site per watch as the response variable. We controlled for variation in hide watch length, by fitting the length (in hours) of each hide watch as an offset in the model. We had multiple watches from the same nest in the same year and therefore to control for this we fitted nest site as a random term in the model. Additionally, to control for the potential effect of brood size, we included brood size as a fixed effect in the model. Linear mixed models were fitted using the lmer function using the lme4 package [4]. Pairwise comparisons (e.g. between chick ages) were made using the difflsmeans functions in the lmerTest package [16]. Denominator degrees of freedom were estimated using the Kenward Rogers methods using the anova function from the lmerTest package [16].

We considered patterns in female begging using the data from the 28 unfed nests. Similar to Ellis et al. [9], we took the unit of analysis as the number of calls per bout, log-transformed to meet parametric requirements of normality. Because our data came from repeated watches at nests and were unbalanced between years, we used Generalised Linear Mixed Models with a unique identifier for nest and year as random terms. Models were fitted with a normal error structure and an identity link function, and all analysis were type III. Using this approach, we explored how intensity of begging differed with chick age (week), and with different brood sizes (as a continuous variable). Explanatory variables were the brood size, the age of the chicks and the polygynous status of the female (monogamous, alpha or beta). We removed all terms that were non-significant at *P* < 0.1. Results for non-significant terms are presented at the point they were removed from the model. Results for the significant terms are presented when they are included in the minimum adequate model (i.e. with all terms significant at <0.1). We took a similar approach to test the relationship between female begging rates and male deliveries during watches.

To test whether the provisioning of supplementary food reduced female begging and male deliveries, we carried out an analysis using only data from 1998 and 1999, when we experimentally fed nests, to compare begging and provisioning rates of males (grams per hour) between fed and control nests. The model structure was the same as above, except we included fed/unfed as a categorical explanatory variable. We were also interested to see whether the pattern of female calling differed between fed and unfed nests in relations to chick age and therefore also included an interaction term between feeding and chick age.

## Results

**Fig. 1:**
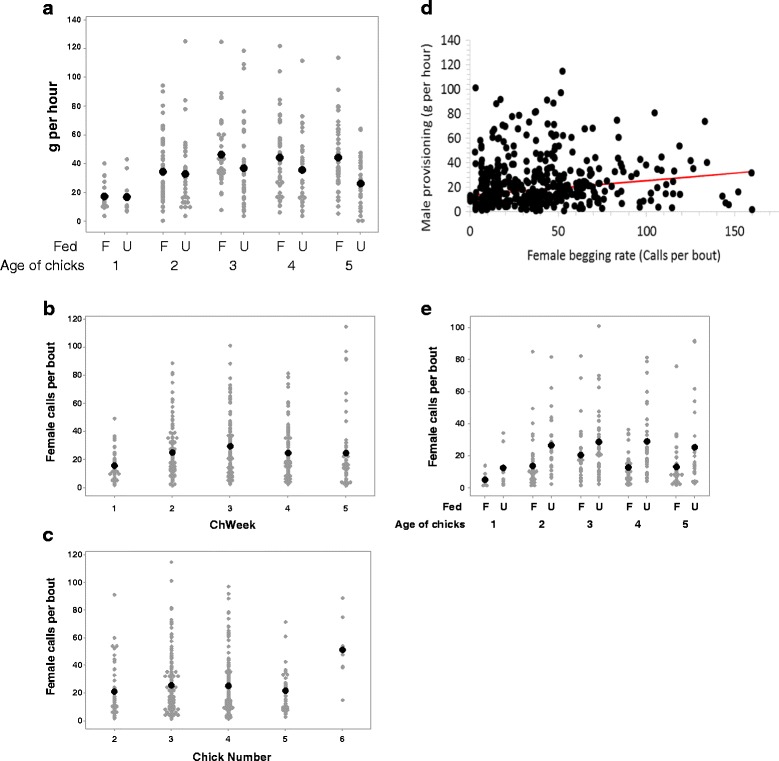
Patterns of food deliveries and female begging at hen harrier nests. **a** shows variation in grams of food delivered to nests with (F) and without (U) access to *adlib* food during the nestling stage (1998 & 1999 data). **b** shows the relationship between female begging rate and the age of chicks in weeks. **c** shows the relationship between female begging rate and brood size. **d** shows the relationship between male provisioning rate (g per hour) and average female begging rate per 6 h watch, with the fitted line showing model output, controlling for chick age and number. **e** shows the effect of experimental feeding on average female begging over the nestling period. Figs show data from individual watches with means (*filled circles*) in **a**, **b**, **c** & **e**

The amount of food delivered by harriers to their young at 14 nests where food was provided *adlib* varied with chick age (χ^2^_4,_ =35.88, *P* < 0.0001, Fig. 1a). The amount of food increased from hatching to three weeks old and then remained similar thereafter with no significant differences between three week old chicks and either four (t ratio = 0.33, *P* = 0.9) or five week old chicks (t ratio = 0.56, *P* = 0.9). A similar pattern was observed the 28 nests without diversionary food. During the years of the feeding experiment, chicks at the 10 nests without diversionary food received less food than chicks at the fed nests (Fig. 1a. F_1, 46.11_ = 12.7, *P* < 0.001).

At the 28 unfed nests, female begging rate varied with chick age (F_4,380_ = 4.85, *P* < 0.001), and showed a similar pattern to the nutritional needs of chicks (Fig. 1b). Begging increased over the first 3 weeks of age, but in this case the rate declined significantly between chicks aged three weeks and chicks aged 5 weeks old (t value = 2.97, df = 379, *P* = 0.007). At these nests there was also a significant positive relationship with brood size (F_1,111_ = 4.95, *P* = 0.027, Fig. 1c). We found no relationship between female begging rate and female breeding status (F_2,23_ = 0.66, *P* = 0.66). After controlling for the significant effects of chick age and brood size, we found a significant positive relationship between male provisioning rates (g per hour) and female begging rate (F_1,385_ = 8.03, *P* < 0.01), although the relationship was weak with considerable scatter (Fig. 1d).

During the feeding experiment in 1998 and 1999 females at nests with food called significantly less than at control nests, throughout the chick rearing period (F_1,57_ = 12.08, *P* < 0.001, Fig. 1e). The pattern of female begging with chick age was broadly similar between the nests with food and the fed and controls, and we found no interaction between chick age and treatment (F_4,277_ = 0.6, *P* = 0.48). There was a tendency for males at nests with food to deliver less g per hour of food than at the control nests in those years, but this was not statistically significant (F_1,21_ = 592.8, *P* = 0.09).

## Discussion

Experimental evidence clearly indicated that begging by female harriers was associated with food intake rate. The availability of *adlib* food at nests greatly reduced the level of female begging. Moreover, our data provided some support for the *Offspring Need Hypothesis*. Female begging rates increased up to the first 3 weeks and then levelled off until fledging, in a pattern similar to the prey intake rate of the chicks. This pattern was the same at both nests with food and controls. In addition, begging rate increased with brood size, again reflecting chick need. We found less support for the *Female Need hypothesis*; females did not increase their calling rate throughout the nestling period, when their hunger levels would be expected to increase.

Secondary females of polygynous pairings in the hen harrier are known to receive less food from their males than monogamous and primary females, but they can compensate for this shortfall by bringing in larger prey items [33]. This compensation may have accounted for the finding that we found no association between female status and begging rates, once the age and size of the brood was accounted for.

Considerable research has investigated how offspring vary their begging investment in relation to their hunger level (reviewed in [22]). Studies have also been carried out to investigate how parents vary their provisioning efforts in relation to the efforts of their partner [21, 24, 35, 38]. In hen harriers, males lack contact with their chicks and so can gain little direct information regarding offspring need. This implies there are increased possibilities in this system for females to manipulate male foraging.

It is widely recognised that communication between males and females should be honest as both have shared interests in reproductive success [40]. Indeed, there are two aspects of this system that suggest that female begging does represent an honest signal of chick need. First, begging calls are likely to be costly. One obvious potential cost lies in increased predation as a result of bouts of loud calling by females in the same way that chick begging can lead to predation [18]. Certainly field workers were guided to nests by the calls of females, implying that predators may similarly benefit. Second, females increased their begging rate when chick needs were greatest, despite this potential cost and they decreased their begging when food was provided. Similar decreases in female begging rate have been found in other systems in response to supplementary food [42, 5, 27].

However, the patterns we observed also suggest that there is some potential for conflict between the sexes. As males and females have a shared interest in fledging their chicks we would expect female begging and male provisioning to have evolved as honest signals [40]. However, we found that male provisioning was only weekly associated with female begging – a pattern in line with other studies which found a weak or no relationship [27, 23, 5]. It is possible that males have less flexibility to increase provisioning rate within seasons [32, 45], whereas females may be able to trade-off increased hunting with time spent at the nest, especially when the chicks are older. We should add one caveat to this discussion. Our data were collected in 6 h watches and it may be that male responses to female begging occur at a different scale.

We also found that females did not stop begging when *adlib* food was provided throughout the nestling period. There are a number of possible alternative explanations for this. First, females may continue to beg, albeit at a lower rate, because of uncertainty in the future environment. The fact that they have ample food today, does not necessarily mean that food will be available tomorrow, so broods need continued investment from the males. Although broods may be currently satiated, the female cannot risk that the male stops providing food in the future, otherwise she will have to spend more time hunting and therefore less time protecting her young [2]. An alternative argument is that females may need to beg at a minimum rate to reinforce male behaviour [37] and ensure continued food provisioning by the male. However, we cannot discount the possibility that female begging rate simply reflected chick begging rate and it is the chicks that are trying to manipulate their parents to keep delivering food, although they are currently satiated.

Future work could measure chick and female begging rates and experiments could focus on manipulating the signals between chicks, females and males. This could be done by feeding chicks directly (rather than providing food via females as we have done here) to reduce their nutritional needs and begging rates, or through using loudspeakers to increase the level of chick begging and/or female begging. Similarly, female need could be manipulated through feeding females during incubation to improve their condition during the early nestling period. Environmental uncertainty could be manipulated by increasing variability in daily food provisioning rates by removing and adding food at nests. These studies should measure responses in male provisioning at different time scales, to see how quickly males can respond to changing begging rates.

## Conclusions

Our analysis of female begging at 42 hen harrier nests provided support for the idea that female begging reflected the needs of their offspring. At nests where we experimentally made food continuously available, females reduced their begging rate, suggesting that begging was an honest signal of need. However, begging continued even with *adlib* food and was only weakly associated with greater provisioning by males, suggesting that these calls may also play an additional role, possibly reflecting sexual or parent-offspring conflict. Further experiments are required to tease out the contribution of these different components.
